# Alpha-1 antitrypsin deficiency in bronchiectasis: Evidence for an overlooked entity beyond COPD: A retrospective observational study

**DOI:** 10.1097/MD.0000000000047298

**Published:** 2026-01-23

**Authors:** Levent Özdemir, Ahmet Cemal Pazarli, Savaş Gegin, Burcu Özdemir, Esra Arslan Aksu, Mahcube Çubukçu

**Affiliations:** a Department of Chest Diseases, Samsun University Faculty of Medicine, Samsun, Turkey; b Department of Pulmonary Diseases, Tokat Gaziosmanpaşa University, Faculty of Medicine, Tokat, Turkey; c Department of Chest Diseases, Samsun Education and Research Hospital, Samsun, Turkey; d Department of Family Medicine, Samsun University Faculty of Medicine, Samsun, Turkey.

**Keywords:** alpha-1 antitrypsin, bronchiectasis, genotype

## Abstract

Alpha-1 antitrypsin deficiency (AATD) is an autosomal co-dominant condition caused by mutations in the SERPINA1 gene. Chronic obstructive pulmonary disease/emphysema, asthma, and bronchiectasis are lung diseases associated with AATD. This study was designed to identify AATD in patients with bronchiectasis without emphysema and to demonstrate the frequency and distribution of AATD genotypes according to the type of bronchiectasis. The study was conducted as a single-center retrospective analysis between December 01, 2022 and December 31, 2024 in patients with bronchiectasis without emphysema. Patients’ demographic characteristics (age, gender), smoking status (smoker, ex-smoker, nonsmoker), and types of bronchiectasis (cylindrical, varicose, cystic) according to the Reid classification were evaluated. Dried blood spot samples collected from fingertip pricks were used to screen for alpha-1 antitrypsin genotype deficiency. A total of 563 patients, 241 (42.8%) women, and 322 (57.2%) men, with bronchiectasis without emphysema were evaluated, with a mean age of 55.3 ± 14.9 years. An AATD mutation was detected in 16 patients (2.8%). Genotype deficiency was most commonly observed in the cylindrical type (n = 9). The most frequently identified genotypes were PI*M malton in 6 patients (1.1%), PI*P lowell in 4 patients (0.8%), and PI*I in 3 patients (0.6%). Additionally, 2 patients were found to have previously unidentified novel alpha-1 antitrypsin variants. One of these patients also had Kartagener syndrome. Our findings suggest an association between AATD and bronchiectasis, independent of emphysema, and suggest that alpha-1 antitrypsin genotypes should also be examined in cases of bronchiectasis without emphysema to determine its etiology.

## 1. Introduction

Alpha-1 antitrypsin deficiency (AATD) is a common but largely underrecognized autosomal co-dominant condition caused by mutations in the SERPINA1 gene, characterized by low serum alpha-1 antitrypsin (AAT) levels.^[1]^ Although AATD is one of the most common hereditary diseases affecting the lungs, it remains underdiagnosed.^[2]^ Chronic obstructive pulmonary disease (COPD)/emphysema, asthma, and bronchiectasis are lung diseases associated with AATD. Therefore, the early diagnosis of AATD is crucial for identifying diseases that can guide physicians toward considering AATD.^[3-5]^

Bronchiectasis is a heterogeneous airway disease characterized by chronic respiratory symptoms and permanent dilation of the bronchi, with increasing prevalence and significant associated mortality and morbidity.^[6]^ While the relationship between AATD and COPD/emphysema is well established, the association between AATD and asthma or bronchiectasis remains a topic of debate.^[1]^

The World Health Organization recommends screening for AATD in patients with COPD and emphysema regardless of smoking history, in asthma patients whose airway obstruction does not fully resolve after bronchodilator therapy, in asymptomatic patients with persistent obstruction on pulmonary function tests and identifiable risk factors, in individuals with siblings carrying the PiZZ allele, and in all patients with necrotizing panniculitis or unexplained liver disease.^[7]^

Although bronchiectasis is one of the treatable diseases associated with AATD, the relationship between bronchiectasis and AATD has not been sufficiently studied. Significant gaps remain regarding whether AATD is an independent factor for the development of bronchiectasis and what clinical effects it may have on disease progression, epidemiology, and treatment. Additionally, patient characteristics have not been well defined.^[8,9]^

This study was designed to identify AATD in patients with bronchiectasis without emphysema and to demonstrate the frequency and distribution of AATD genotypes according to the type of bronchiectasis.

## 2. Materials and methods

**Figure 1.**
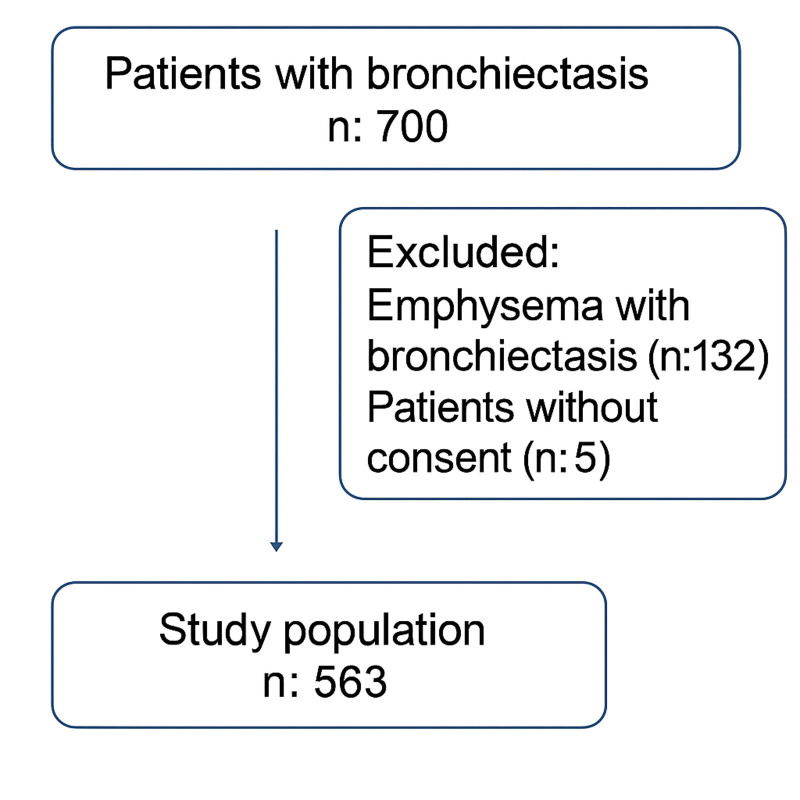
Study design.

The study was conducted as a cross-sectional descriptive study in the pulmonary diseases clinic of Samsun Training and Research Hospital between December 1, 2022 and December 31, 2024, by evaluating the high-resolution thoracic computed tomography (HRCT) scans of 700 patients with bronchiectasis. A total of 563 bronchiectasis patients without emphysema on HRCT were included in the study (Fig. 1). Patients were included retrospectively by reviewing the medical records of our center. All eligible cases were assessed according to predefined inclusion and exclusion criteria, and no consecutive enrollment was performed.

### 2.1. Inclusion criteria

Patients over 18 years of age who provided informed consent, agreed to participate in the study, and were diagnosed with bronchiectasis without emphysema on high-resolution thoracic computed tomography.

### 2.2. Exclusion criteria

Patients with bronchiectasis and emphysema on high-resolution thoracic computed tomography and patients who did not consent to provide fingertip blood samples for the study, and those with incomplete demographic, HRCT, or genotyping data were excluded from the study.

The study complies with the Declaration of Helsinki and approval for the study was obtained from the Samsun University Non-Interventional Clinical Research Ethics Committee (Date: 08.01.2025, Decision No. 2025/1/11). Written informed consent was obtained from the patients after providing them with information about their condition.

Based on the relevant literature, the AAT genotyping test is used to simultaneously identify the 14 most common allele variants associated with AAT deficiency. This test is based on the amplification of genomic DNA using polymerase chain reaction and subsequent hybridization with allele-specific probes utilizing Luminex xMAP technology. Dried blood spot samples collected from the fingertips were used to screen for alpha-1 antitrypsin (AAT) genotype deficiency in patients with bronchiectasis. The validation of the novel variants was confirmed by DNA sequencing at the Progenika reference laboratory in Spain. The genotype analysis (AlphaKits^®^ GE Healthcare Ltd., Cardiff, CF147YT, UK) was performed at the Progenika clinical diagnostics laboratory in Spain. The alleles examined in the patients included PI*I, PI*M procida, PI*M malton, PI*S iiyama, PI*Q0 granite falls, PI*Q0 west, PI*Q0 bellingham, PI*F, PI*P lowell, PI*S, PI*Z, PI*Q0 mattawa, PI*Q0 clayton, and PI*M heerlen. All 563 cases had complete core data (demographics, HRCT, genotyping); no imputation was required.

**Figure 2.**
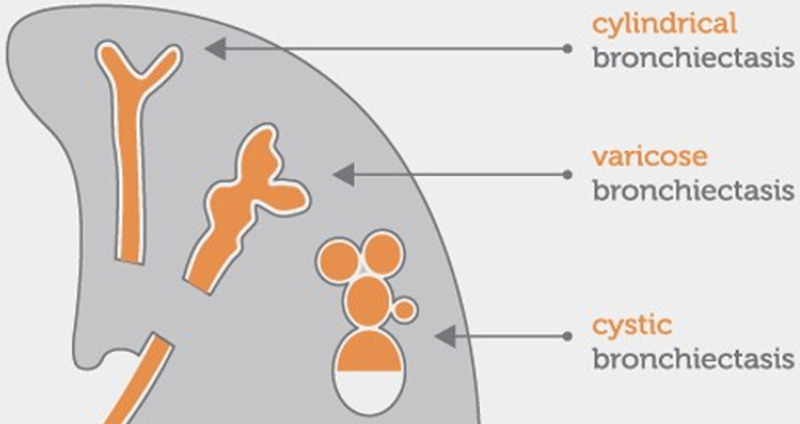
Bronchiectasis types.

In the screening, patients’ demographic characteristics (age, gender), smoking status (smoker, ex-smoker, nonsmoker), and bronchiectasis types based on the Reid classification (cylindrical, varicose, cystic) were recorded (Fig. 2).

Bronchiectasis types (Fig. 2)^[10]^:

Cylindrical bronchiectasis: The bronchi are enlarged and cylindrical.
Varicose bronchiectasis: The bronchi are irregular with alternating areas of dilation and constriction.
Cystic bronchiectasis: Dilated bronchi form clusters of cysts.

The alpha-1 antitrypsin levels and pulmonary function tests of patients with detected alpha-1 antitrypsin genotype deficiency were evaluated. The alpha-1 antitrypsin levels and pulmonary function tests were conducted when the patients were not experiencing an exacerbation, and their CRP levels were within the normal range. Serum AAT levels were measured in a single accredited laboratory using the same standardized nephelometric assay and reference range for all samples.

## 3. Statistical analysis

All analyses were performed via SPSS 22 for Windows program (SPSS Inc., Chicago). Frequencies and percentages of categorical variables and mean, median, and standard deviation values of numerical variables were calculated.

## 4. Results

Between December 1, 2022 and December 31, 2024, a total of 563 bronchiectasis patients without emphysema were evaluated, with a mean age of 55.3 ± 14.9 years (male: 56.4 ± 13.8, female: 53.9 ± 16.1). Of these patients, 241 (42.8%) were female and 322 (57.2%) were male. Smoking status was distributed as follows: 169 (30%) were current smokers, 300 (53.3%) were nonsmokers, and 94 (16.7%) were ex-smokers. Among patients with bronchiectasis, the cylindrical type was the most common (n = 320, 56.8%; Table 1).

**Table 1:** Demographic characteristics of bronchiectasis cases without emphysema who were screened for alpha-1 antitrypsin genotype deficiency.

Gender	Female n (%) 241 (42.8)	Male n (%) 322 (5.2)	Total n 563
Age	53.9 ± 16.1	56.4 ± 13.8	55.3 ± 14.9
Smoking status			
Nonsmoker	209 (37.1)	91 (16.2)	300 (53.3)
Smoker	16 (2.8)	153 (27.2)	169 (30)
Ex-smoker	16 (2.8)	78 (13.9)	94 (16.7)
Bronchiectasis type			
Cylindrical	123 (21.8)	197 (35)	320 (56.8)
Varicose	30 (5.3)	29 (5.2)	59 (10.5)
Cystic	88 (15.6)	96 (17.1)	184 (32.7)

In the AAT genotyping results of the patients, no mutations were detected in 547 patients (97.2%), while AATD mutations were identified in 16 patients (2.8%). The genotype distribution according to the type of bronchiectasis is shown in Table 2.

**Table 2:** Distribution of genotypes according to bronchiectasis type.

Genotip	Cylindrical n (%)	Varicose n (%)	Cystic n (%)
PI*MM	311 (55.2)	57 (10.1)	179 (31.8)
PI*M malton	6 (1.1)	0	0
PI*P lowell	2 (0.4)	0	2 (0.4)
PI*I	1 (0.2)	1 (0.2)	1 (0.2)
PI*Z	0	1 (0.2)	0
Undefined	0	0	2 (0.4)
GRCh38 (g.94382987)	0	0	1 (0.2)
GRCh38 (g.94388565)	0	0	1 (0.2)

The characteristics of patients with defects in the SERPINA1 gene, based on genotype distribution, are presented in Table 3. Genotype deficiency was most commonly detected in the cylindrical type (n = 9). Of the patients with detected mutations, 10 were female and 6 were male. Eleven patients were nonsmokers, while 1 patient was a smoker. The most frequently detected genotypes with mutations were PI*M malton in 6 patients (1.1%), PI*P lowell in 4 patients (0.8%), and PI*I in 3 patients (0.6%). A previously unidentified alpha-1 Antitrypsin variant was detected in 2 patients. One of these patients was also diagnosed with Kartagener syndrome. The serum AAT levels of patients with identified AATD alleles were 1.20 ± 0.27 g/L. In pulmonary function tests, the mean forced expiratory volume in 1 second was 1.88 ± 0.87 L (62.3 % ± 22.6), and forced expiratory volume in 1 second was <70% in 7 patients.

**Table 3:** Characteristics of patients with defects in the SERPINA1 gene according to genotype distribution.

Genotip	Age gender	Cigarete	Bronchiestasis type	AAT level N: 0.9–2 g/L	FVC L (%)	FEV_1_ L (%)	FEV_1_/FVC (%)
PI*M malton							
Case 1	Y/M	Ex-smoker	Cylindrical	1.39	1.56 (42)	1.14 (38)	90
Case 2	MA/M	Ex-smoker	Cylindrical	1.42	2.09 (67)	2.03 (82)	121
Case 3	Y/M	Smoker	Cylindrical	0.99	2.23 (58)	1.46 (47)	81
Case 4	Y/M	Nonsmoker	Cylindrical	1.8	2.45 (72)	2.62 (83)	93
Case 5	Y/F	Nonsmoker	Cylindrical	1.39	2.68 (84)	3.26 (81)	103
Case 6	Y/F	Nonsmoker	Cylindrical	0.6	2.10 (67)	1.70 (64)	102
PI*P lowell							
Case 7	Y/F	Nonsmoker	Cystic	1.06	3.63 (71)	3.42 (82)	115
Case 8	Y/F	Nonsmoker	Cylindrical	1.29	2.14 (92)	2.32 (82)	111
Case 9	Y/M	Ex-smoker	Cylindrical	1.06	2.86 (70)	2.66 (82)	117
Case 10	O/F	Ex-smoker	Cystic	1.51	1.04 (30)	0.95 (35)	115
PI*I							
Case 11	MA/F	Nonsmoker	Cylindrical	0.97	1.45 (61)	1.35 (73)	115
Case 12	Y/F	Nonsmoker	Varicose	1.20	2.05 (82)	2.14 (72)	113
Case 13	Y/F	Nonsmoker	Cystic	1.19	2.28 (86)	2.3 (74)	99.1
PI × M/Z							
Case 14	MA/M	Nonsmoker	Varicose	0.99	1.35 (73)	1.45 (61)	93.1
Undefined							
Case 15	Y/F	Nonsmoker	Cystic	1.1	0.81 (30)	0.84 (26)	112
Case 16	Y/F	Nonsmoker	Cystic	1.21	0.36 (13)	0.36 (16)	125

AAT = alpha-1 antitrypsin, F = female, FEV₁ = forced expiratory volume in 1 second, FEV₁/FVC = ratio of forced expiratory volume in 1 second to forced vital capacity, FVC = forced vital capacity, M = male, MA = middle age (66–79 age), O = old (>80 age), Y = young (18–65 age).

## 5. Discussion

This cross-sectional descriptive study was designed to identify patients with mutations in the SERPINA1 gene, a potential genetic cause of bronchiectasis, in patients with bronchiectasis without emphysema. It demonstrated that AATD was detected in 2.8% of patients with bronchiectasis without emphysema.

Although bronchiectasis is recognized as a feature in some patients with alpha-1 antitrypsin deficiency, there is limited literature on AAT genotypes in patients with bronchiectasis. The prevalence and patient characteristics are not widely known. Studies have shown that both the frequency of bronchiectasis in patients with AATD and the prevalence of AATD in patients with bronchiectasis have been evaluated. To the best of our knowledge, our study is the first to report genotypic AATD screening results in patients with bronchiectasis without emphysema.

Bronchiectasis is observed in patients with AATD; however, it is unclear whether there is an independent association with COPD. In the first report by EARCO, which evaluated the prevalence of bronchiectasis in patients with alpha-1 antitrypsin deficiency, emphysema and bronchiectasis were detected in 113 out of 418 patients (27%) with the PiZZ genotype, while bronchiectasis alone was observed in 38 patients (9.1%).^[11]^ In a study by Soyza et al, bronchiectasis was identified independently of COPD in 29.9% of patients with the PiZZ genotype, 8.3% with the PiSZ genotype, and 8.6% with the PiMZ genotype.^[9]^ These 2 studies support the existence of an independent relationship between AATD and bronchiectasis, separate from emphysema and COPD.

In a study of 475 patients with AATD, bronchiectasis was identified in 58 (12%) patients (PiZZ: 43, PiSZ: 4, others: 11).^[12]^ Similarly, Shin et al found bronchiectasis in 3 of 7 patients with the homozygous PiZZ phenotype.^[13]^ Neither study distinguished patients with COPD/emphysema.

In a screening study by Onur et al including 1087 patients with COPD, asthma, and bronchiectasis, AATD mutations were detected in 2.2% (n = 5) of 225 bronchiectasis patients.^[14]^ Lonni et al reported AATD in 0.6% (n = 8) of 1258 bronchiectasis cases.^[15]^ Genotype frequencies for bronchiectasis were not separately reported in either study.

In the study by Veith et al, evaluating 18,736 patients with COPD/emphysema, asthma, and bronchiectasis, 285 bronchiectasis patients (1.52%) showed the following genotype distribution: PiMM 59.3%, PiMZ 21.4%, PiZZ 7.02%, PiMS 4.91%, PiSZ 2.81%, PiM/Rare 2.11%, PiSS 0.7%, PiS/Rare 0.7%, PiRare/Rare 0.7%, and PiZ/Rare 0.35%.^[1]^

Pasteur et al reported PiMM, PiMS, PiMZ, and PiSS phenotypes in 83%, 9.3%, 7.3%, and 0.6% of 150 bronchiectasis patients, respectively.^[16]^ Another study analyzing 285 bronchiectasis patients found PiMM 92.3%, PiMS 4.9%, PiMZ 1.8%, PiSS 0.4%, and PiZZ 0.7%.^[17]^

In our study, AATD was identified in patients with bronchiectasis without emphysema, and these results are consistent with the findings of EARCO’s first report and the study by Soyza et al The EARCO registry included patients with both emphysema and bronchiectasis, whereas our study exclusively enrolled bronchiectasis patients without emphysema, confirmed by HRCT. In addition, while De Soyza et al evaluated a heterogeneous population comprising PiZZ, PiSZ, and PiMZ individuals, our analysis focused on a Turkish cohort, using genotypic screening that covered 14 alleles. When examining studies conducted on patients with bronchiectasis, a higher rate of AATD (2.8%) was detected compared to the data in the literature. Unlike other studies, our study also examined AAT genotyping based on the type of bronchiectasis. The PI*M malton genotype (n = 6) was more frequently detected in patients with cylindrical bronchiectasis (n = 9).

The benefits of augmentation therapy are well established for COPD and emphysema related to AATD; however, its role in bronchiectasis without emphysema remains uncertain. To date, no studies have shown clinical improvement or slowed disease progression in such patients, although isolated case reports suggest potential benefit. Accordingly, screening bronchiectasis patients for genetic disorders is important to identify candidates who may benefit. Current European Respiratory Society and British Thoracic Society guidelines do not recommend AATD screening in bronchiectasis without emphysema.^[18,19]^ In our cohort, although 7 patients had reduced pulmonary function, their AAT levels were within the normal range. One patient had both reduced AAT levels and impaired function but did not receive augmentation therapy due to lack of indication.

This study has several limitations. First, it was conducted as a single-center, retrospective study, which may limit the generalizability of the findings to broader populations. Second, the absence of a control group prevents direct comparison with patients having bronchiectasis of other etiologies or with emphysema. Third, the study design did not allow for longitudinal follow-up to assess clinical outcomes or disease progression. Given the descriptive aim (prevalence, distribution), no inferential or sensitivity analyses were planned; this is acknowledged as a study limitation.

In conclusion, none of the bronchiectasis patients in whom we identified AATD had emphysema on HRCT. Our findings suggest a possible association between AATD and bronchiectasis independent of emphysema. We believe that AAT genotyping should also be considered in the evaluation of bronchiectasis etiology in cases of bronchiectasis without emphysema, as AAT deficiency is a treatable condition.

## Author contributions

**Conceptualization:** Levent Özdemir, Ahmet Cemal Pazarli.

**Data curation:** Levent Özdemir, Savaş Gegin.

**Formal analysis:** Levent Özdemir, Ahmet Cemal Pazarli.

**Funding acquisition:** Savaş Gegin.

**Methodology:** Savaş Gegin, Esra Arslan Aksu.

**Project administration:** Levent Özdemir, Ahmet Cemal Pazarli, Burcu Özdemir.

**Resources:** Savaş Gegin, Burcu Özdemir, Esra Arslan Aksu.

**Software:** Burcu Özdemir, Esra Arslan Aksu.

**Supervision:** Levent Özdemir, Esra Arslan Aksu.

**Validation:** Ahmet Cemal Pazarli, Mahcube Çubukçu.

**Visualization:** Ahmet Cemal Pazarli, Savaş Gegin, Mahcube Çubukçu.

**Writing – original draft:** Levent Özdemir, Ahmet Cemal Pazarli.

**Writing – review & editing:** Levent Özdemir, Ahmet Cemal Pazarli.
